# Changes in Topical Corticosteroid Dispensing Patterns Following Specialist Consultation in Patients with Atopic Dermatitis: A Two-year Longitudinal Study

**DOI:** 10.2340/actadv.v106.adv-2026-0783

**Published:** 2026-10-06

**Authors:** Fatema Shahin, Anne Sofie Frølunde, Christian Vestergaard

**Affiliations:** 1 Department of Dermatology and Venereology, Aarhus University Hospital, Aarhus, Denmark; 2 Department of Clinical Medicine, Aarhus University, Aarhus, Denmark

Atopic dermatitis (AD) is a prevalent chronic in-flammatory skin disease with a substantial psychosocial and clinical burden, affecting patients across all ages and ethnicities (1).

Topical corticosteroids (TCS) remain the first-line treatment for mild AD and the management of acute flare-ups across all severities and are essential for achieving and maintaining adequate disease control (2). TCS efficacy depends on correct potency, sufficient quantity and patient education (3). In Europe, TCS are categorized into 4 potency classes and should be prescribed according to disease severity and the anatomical location of the eczema (4). Despite esta-blished treatment guidelines, evidence suggests that patients with AD are often undertreated in primary care (5–7). Contributing factors may include corticophobia among patients and healthcare professionals and limited guidance in appropriate TCS use (8–10).

Clinical observations suggest that a subset of patients referred for specialist care has not received optimized topical therapy prior to their referral (11). Consequently, this study investigated patterns of TCS use during the year preceding referral and the year following the initial specialist consultation. Specifically, we focused on changes in prescribed potency and quantity to evaluate changes in treatment following specialist intervention.

## MATERIALS AND METHODS

This retrospective registry-based study was conducted at the Department of Dermatology and Venereology, Aarhus University Hospital. The project was approved according to institutional regulations, and all patients provided consent for access to medical data. Formal ethical approval was not required.

Patients referred with a diagnosis of AD between 2021 and 2023 were screened for eligibility. From a list of 460 referred patients with AD, consecutive patients were contacted until the predefined study population was reached. Patients referred outside the study period or without a confirmed AD diagnosis were excluded.

Data on TCS prescriptions redeemed at pharmacies were obtained from the Danish Shared Medication Record. TCS use was assessed during the year prior to referral and the year following the first hospital consultation. Patients were stratified into predefined age groups: 0–5, 6–12, 13–18 and ≥18 years. TCS products were categorized according to the European potency classification as group I–IV: mild, moderate, potent, and very potent.

Demographic and clinical variables, including age, sex, referring physician specialty, atopic comorbidities and treatment initiated at first consultation, were extracted from electronic medical records. TCS con-sumption was calculated in grams based on dispensed prescriptions.

### Statistical analysis

Mean dispensed TCS before and after the first consultation was compared using paired individual-level analyses. Patients with no recorded dispensing of a given TCS potency class were coded as having zero grams for that class, consistent with the number of patients reported for each age group. Normality of the paired differences was assessed using the Shapiro-Wilk test, which indicated non-normal distribution in the majority of age- and potency-stratified subgroups. The Wilcoxon signed-rank test was therefore used for all paired comparisons, and exact p-values are reported given the small subgroup sizes and the high frequency of tied (zero) values.

Statistical significance was defined as *p*<0.05. Analyses were performed using StataNow/MP version 19.5 (StataCorp LLC).

## RESULTS

A total of 119 patients were included. Overall, age at first consultation ranged from 2 months to 84 years. Median age ranged from 2 years (IQR 0.9–3.3) in the youngest age group to 39 years (IQR 25.9–50.8) among adults. In the year prior to referral, 33.6% of patients (95% CI 25.6–42.6%) had no recorded dispensed TCS (**Table I**).

**Table I.** Use of TCS prior to first hospital consultation

	*N* (%)	95% CI
Use of topical corticosteroid prior	79 (66.39)	0.57–0.74
No use of topical corticosteroid prior	40 (33.61)	0.26–0.43

Following specialist consultation at the hospital, overall TCS use increased compared with the year preceding referral. When stratified by the predefined age groups, increases were observed across all groups. Among preschool children aged 0–5 years significant increases were observed for moderately potent TCS (group II) (+446.6%, *p*=0.001) and potent TCS (group III) (+719.2%, *p*<0.001). No comparison reached statistical significance in the 6–12 or 13–18 age groups. Among adults aged ≥18 years, significant increases were observed across all 4 potency classes: mild TCS (group I) (+223.3%, *p*=0.024), moderately potent TCS (group II) (+823.5%, *p*<0.001), potent TCS (group III) (+197.1%, *p*=0.015) and very potent TCS (group IV) (+386.0%, *p*=0.026). At the first hospital consultation, 54.6% of patients were managed with topical treatment (and patient education) alone, whereas 40.3% required initiation of systemic therapy (**Table II**).

**Table II.** Choice of treatment at the first consultation in the hospital after referral

	*N*	%
Topical treatment	65	54.62
Systemic treatment	48	40.34
No treatment	2	1.68
Other treatment	4	3.36
Total	119	100

## DISCUSSION

This study found that 33.6% of patients with AD referred to the outpatient clinic had no dispensed TCS in the year prior to referral, while the overall amount of dispensed TCS increased after referral. Notably, more than half of the patients were managed with optimized topical treatment alone after dermatologist consultation at the hospital, suggesting that topical therapy had not been fully optimized prior to referral in a subset of the patients. This may reflect undertreatment in primary care for some patients, potentially related to insufficient prescribing of TCS potency and/or quantity, reluctance to use TCS due to concerns about adverse effects, or limited knowledge regarding appropriate use.

Previous studies have described corticophobia among both patients and healthcare professionals (8–10, 12). Our findings support these observations and suggest that inadequate TCS prescribing and insufficient patient education may contribute to undertreatment of AD in primary care setting. This is clinically relevant given the long waiting times for specialist dermatology care.

Of the 16 subgroup comparisons presented in **Table III**, 6 reached statistical significance. Since only ~1 significant result would be expected by chance across 16 comparisons at α=0.05, multiple testing alone is unlikely to explain these findings. Nonetheless, given the number of comparisons performed and the small size of some subgroups, individual results close to the significance threshold should be interpreted with caution.

**Table III.** Mean amount of dispensed topical corticosteroids (TCS) 1 year before and 1 year after first hospital consultation, stratified by age group and potency

Age group	TCS potency	1 year prior Mean (g)	1 year after Mean (g)	Difference (g)	Change (%)	*p*-value
0–5 years (*n*=35)	Mild	8.3	5.4	-2.9	−34.5	0.813
	Moderate	20.9	114.0	+93.1	+446.6	**0.001**
	Potent	16.3	133.4	+117.1	+719.2	**<0.001**
	Very potent	0.0	5.7	+5.7	-	1.000
6–12 years (*n*=12)	Mild	0.0	2.5	+2.5	-	1.000
	Moderate	0.0	43.3	+43.3	-	0.125
	Potent	25.0	23.3	-1.7	−6.7	1.000
	Very potent	0.0	5.0	+5.0	-	1.000
13–18 years (*n*=6)	Mild	0.0	0.0	0.0	-	-
	Moderate	0.0	15.0	+15.0	-	1.000
	Potent	31.7	170.0	+138.3	+436.8	0.250
	Very potent	0.0	0.0	0.0	-	-
>18 years (*n*=66)	Mild	3.3	10.5	+7.3	+223.3	**0.024**
	Moderate	7.7	71.4	+63.6	+823.5	**<0.001**
	Potent	46.4	137.7	+91.4	+197.1	**0.015**
	Very potent	7.6	36.8	+29.2	+386.0	**0.026**

The *p*-values were calculated using the wilcoxon signed-rank test (exact *p*), as Shapiro–Wilk testing of the paired differences indicated non-normal distributions in most subgroups. Percentage change was not calculated when baseline use was zero.

However, several limitations should be considered when interpreting these findings. First, the study was observational and retrospective, without a primary-care comparator group; the observed increase in TCS dispensing after referral should therefore be interpreted with caution regarding causality.

Second, patients were recruited using systematic sampling from the full list of referred patients, and no formal power calculation was performed; the relatively small size of some age strata (13–18 years, *n*=6; 6–12 years, *n*=12) may therefore limit statistical power. Although the sampling approach was largely independent of clinical characteristics, patients lost to death, nonresponse or refusal during recruitment were not systematically compared with participants, and we cannot exclude the possibility that these patients may differ in disease severity or treatment history. Finally, dispensed TCS does not necessarily reflect actual use or adherence, and individual consumption cannot be assessed with complete accuracy, as products may be shared within households, potentially leading to misestimation. Despite these limitations, the dispensing data provide an objective estimate of treatment patterns and are consistent with clinical observations from everyday practice.

In conclusion, TCS dispensing increased in the year following the first hospital consultation, and topical treatment appears to not have been fully optimized prior to referral in a substantial proportion of patients with AD. As this study was retrospective and observational, without a comparator group, the observed increase cannot be attributed with certainty to the specialist consultation itself. Nonetheless, improved knowledge of TCS use and optimization of topical treatment in primary care could potentially reduce unnecessary referrals and waiting times, allowing specialist care to focus on patients requiring systemic treatment.

## Data Availability

The data that support the findings of this study are available from the corresponding author upon reasonable request.
